# Curdione ameliorates bleomycin-induced pulmonary fibrosis by repressing TGF-β-induced fibroblast to myofibroblast differentiation

**DOI:** 10.1186/s12931-020-1300-y

**Published:** 2020-02-19

**Authors:** Peng Liu, Kang Miao, Lei Zhang, Yong Mou, Yongjian Xu, Weining Xiong, Jun Yu, Yi Wang

**Affiliations:** 1 Department of Respiratory and Critical Care Medicine, Key Laboratory of Pulmonary Diseases of Health Ministry, Key Cite of National Clinical Research Center for Respiratory Disease, Wuhan Clinical Medical Research Center for Chronic Airway Diseases, Tongji Hospital, Tongji Medical College, Huazhong University of Sciences and Technology, 1095 Jiefang Ave, Wuhan, 430030 China; 2 Department of Respiratory Medicine, Shanghai Ninth People’s Hospital, Shanghai Jiaotong University School of Medicine, 639 Zhizaoju Lu, Shanghai, 201999 China; 3 Department of Thoracic Surgery, Tongji Hospital, Tongji Medical College, Huazhong University of Sciences and Technology, 1095 Jiefang Ave, Wuhan, 430030 China

**Keywords:** IPF, Fibroblast, Myofibroblast, Curdione, TGF-β1

## Abstract

**Background:**

Idiopathic pulmonary fibrosis (IPF) is a progressive and irreversible disease characterized by excessive fibroblast to myofibroblast differentiation with limited therapeutic options. Curdione, a sesquiterpene compound extracted from the essential oil of *Curcuma aromatica* Salisb, has anti-inflammatory and anti-tumor effects. However, the role of curdione in IPF is still unclear.

**Methods:**

The effects of curdione were evaluated in a bleomycin (BLM)-induced pulmonary fibrosis mouse model. C57BL/6 mice were treated with BLM on day 0 by intratracheal injection and intraperitoneal administered curdione or vehicle. In vitro study, expression of fibrotic protein was examined and the transforming growth factor (TGF)-β-related signaling was evaluated in human pulmonary fibroblasts (HPFs) treated with curdione following TGF-β1 stimulation.

**Results:**

Histological and immunofluorescent examination showed that curdione alleviated BLM-induced lung injury and fibrosis. Specifically, curdione significantly attenuated fibroblast to myofibroblast differentiation in the lung in BLM induced mice. Furthermore, curdione also decreased TGF-β1 induced fibroblast to myofibroblast differentiation in vitro*,* as evidenced by low expression of α-SMA, collagen 1 and fibronectin in a dose dependent manner. Mechanistically, curdione suppressed the phosphorylation of Smad3 following TGF-β1 treatment, thereby inhibiting fibroblast differentiation.

**Conclusions:**

Overall, curdione exerted therapeutic effects against pulmonary fibrosis via attenuating fibroblast to myofibroblast differentiation. As curdione had been shown to be safe and well-tolerated in BLM-induced mouse model, curdione might be useful for developing novel therapeutics for IPF.

## Introduction

Idiopathic pulmonary fibrosis (IPF) is the most common type of idiopathic interstitial pneumonia, with a five-year survival rate of less than 30% [1]. Previous studies have demonstrated that the pathogenesis of IPF involves the repair of lung tissue injury by alveolar epithelial cells, the differentiation and proliferation of fibroblasts, and the recruitment and activation of immune cells [2]. Although substantial efforts have recently been devoted to the treatment of IPF, effective treatment options still remain limited [3]. Therefore, discovering new therapeutic drugs and demonstrating their efficacy and safety are priority needs for IPF patients.

Previous studies have consistently demonstrated that curdione, a sesquiterpene compound extracted from the essential oil of *Curcuma aromatica* Salisb [4], possesses beneficial properties against inflammation [5], oxidative Stress [6], tumor growth [7] and fungal infection [8]. More interestingly, there is feasible evidence suggesting the potential role of curdione to attenuate the proliferation of hepatic myofibroblast cells [9]. As is known, both inflammatory response and myofibroblasts play vital roles in the pathological process of IPF. These findings prompted us to hypothesize that curdione may be a good candidate drug for treatment of IPF.

To address this feasibility, we induced pulmonary fibrosis in mice and then assessed the impact of curdione on the disease development. Remarkably, treatment with curdione provided protection for mice against BLM-induced pulmonary fibrosis as manifested by attenuated lung injury and fibrosis, and markedly reduced fibroblast to myofibroblast differentiation in the lung. In line with this observation, curdione also decreased the expression level of fibronectin, collagen 1 and α-SMA in TGF-β1 stimulated HPFs and significantly inhibited TGF-β/Smad3 signaling. Together, our studies suggest that administration of curdione might be a viable approach to the treatment of IPF in clinical settings.

## Material and methods

### Animals

Sixty male C57BL/6 mice (8 weeks old) were obtained from the Beijing Vital River Laboratory Animal Technology Co., Ltd. (Beijing, China) and housed in a specific pathogen-free (SPF) animal facility at the Tongji Medical College with sterile acidified water and irradiated food. The Institutional Animal Care and Use Committee of Tongji Hospital (Wuhan, China) approved the protocols used for the animal experiments in the present study.

### Reagents and antibodies

Curdione was purchased from Shanghai yuanye Bio-Technology Co., Ltd. (Shanghai, China), and Bleomycin was obtained from MedChemExpress LLC (NJ, USA). Antibodies against β-actin, Gapdh, α-SMA, fibronectin, collagen I, p-Jnk, Jnk, p-p38, p38, p-Erk1/2, Erk1/2, p-Smad2, p-Smad3, Smad2/3 were purchased from Cell Signal Technology Inc. (MA, USA). BCA Protein Assay Kit was supplied by Boster Biological Technology co. Ltd. (Wuhan, China). RT-PCR assay kit was obtained from Takara (Dalian, China). Hydroxyproline assay kit was obtained from Nanjing Jiancheng Bioengineering Institute (Nanjing, China). Cell Counting Kit-8 was available from DojindoChemical technology co. LTD (Shanghai, China).

### Animal treatments

**Fig. 1:**
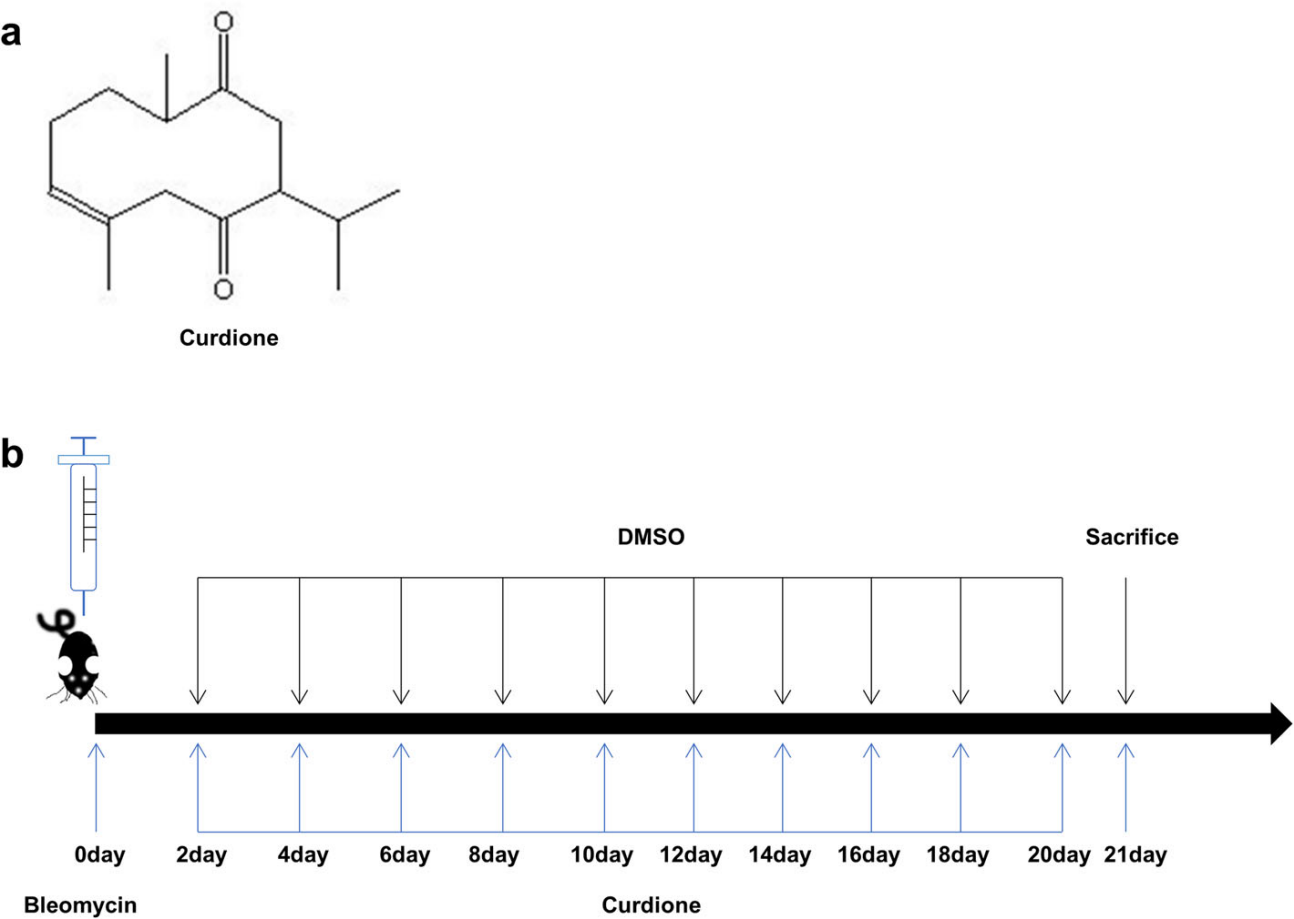
Time course of BLM and curdione administration. **a** Chemical structure of curdione. **b** mice were anesthetized with pentobarbital sodium (80 mg/kg) and instilled intratracheally with BLM (1.75 mg/kg). And then mice were treated intraperitoneally with DMSO or curdione for every two days following BLM injection

All animals were divided randomly into four groups: (i) PBS group, (ii) DMSO group, (iii) BLM + DMSO group, (iiii) BLM + curdione (BLM + CUR) group. For BLM + DMSO group or BLM + CUR group, mice were anesthetized with intraperitoneal injection of pentobarbital sodium (60 mg/kg) and then intratracheally administered 1.75 mg/kg BLM in sterile PBS using established techniques [10]. Next, mice were treated intraperitoneally with curdione (100 mg/kg, dissolved in 10%DMSO) or 10%DMSO every 2 days following BLM injection. Mice were sacrificed under anesthesia on the 21st day (Fig. 1 b).

### Cell culture

HPFs were purchased from ScienCell Research Laboratories, Inc. (Nanjing, China). HPFs were cultured in fibroblast medium supplemented with 10% fetal calf serum,1% FGS and 1% solution of penicillin and streptomycin at 37 °C in a humid atmosphere containing 5% CO_2_. HPFs were treated with stimulated with curdione (160 μM or 300 μM) or equal amount of DMSO 1 h before stimulation with human recombinant TGF-β1 (10 ng/ml, PeproTech, NJ, USA) for 48 h.

### Cell viability assay

Cell viability was assayed by the Cell Counting Kit-8. Briefly, cells were seeded in 96-well plates at 4 × 10^3^ cells per well, then subjected to serum starvation for 24 h by reducing FBS to 2%. Next, the cells were treated different concentrations of curdione (100 μM, 200 μM, 300 μM, 400 μM, 500 μM) or 2‰ of DMSO for 1 h and 48 h, and then 5 mg/ml CCK8 was added to the wells and incubated for additional 2 h. After mix well, the optical density was measured at 450 nm.

### Determination of lung hydroxyproline

Lung specimens were washed with PBS and hydrolyzed with 0.6% hydrochloric acid at 95 °C for 5 h. Hydrolysates were neutralized with sodium hydroxide and diluted with distilled water. Hydroxyproline level in hydrolysates was colorimetrically determined by absorbance at 550 nm with p-dimethylaminobenzaldehyde and expressed as μg/mg wet tissue.

### Histological and immunohistochemical analysis

The left lungs were fixed by intratracheal infusion of 4% aqueous buffered formalin for 24 h, then subjected to paraffin embedding and sliced into 4 μm sections. After dewaxing, the sections were subjected to hematoxylin and eosin (H&E), Sirius red and Masson staining using established techniques [10]. The severity of lung fibrosis was assessed by two pathologists using a brightfield (Olympus, Tokyo, Japan) in a blinded fashion via Ashcroft scoring system. For immunostaining, after dewaxing to water, the sections were co-incubated with primary antibodies against CD3 (1:100) or F4/80(1:100), followed by probing with Horseradish peroxidase labeled secondary antibodies.

### Real-time PCR

Total RNA was isolated using TRIzol reagent (Invitrogen, CA, USA). RNA quantity and quality were measured using the NanoDrop 2000 spectrophotometer (Thermo scientific, MA, USA). Complementary DNA synthesis was performed using the M-MLV reverse transcriptase kit (Invitrogen, CA, USA) as previous reported [11]. RT-PCR was performed using a SYBR green-based PCR master mix kit (Takara, Dalian, China) on a Rotor Gene 3000 real-time PCR system from Corbett Research (Sydney, Australia). The following primers were used for each target gene: Fibronectin (5′-GAT GTC CGA ACA GCA GCT TAT TTA CCA-3′, 5′- CCT TGC GAC TTC AGC CAC T-3′); Collagen 1 (5′- TAA GGG TCC CCA ATG GTG AGA-3′, 5′- GGG TCC CTC GAC TCC TAC AT-3′); α-SMA (5′- GGA CGT ACA ACT GGT ATT GTG C -3′, 5′- TCG GCA GTA GTC ACG AAG GA-3′); and β-actin (5′- GCC ACA GCA CTC CAT CGA C-3′, 5′-GTC TCC GAT CTG GAA AAC GC-3′).

### Western blot analysis

Lung tissues and cultured cells were homogenized in RIPA lysis buffer supplemented with a protease inhibitor cocktail, as previously described [12]. The proteins were subjected to western blotting with the indicated primary antibodies and detected by chemiluminescencec (Advansta, CA, USA).

### Immunofluorescence analysis

For the Immunofluorescence assay, the slides were first probed with Fibronectin or Collagen 1 or α-SMA antibody at 4 °C overnight. After rinsed with PBS, cells were incubated with Alexa 488-conjugated anti-rabbit antibody (Invitrogen, CA, USA), as previously described [13]. The sections were immediately subjected to fluorescence analysis under a fluorescence microscope.

### Assessment of liver function and renal function

Liver function and renal function was assessed by measuring blood Alanine aminotransferase, Aspartate aminotransferase, urea and serum creatinine at the Department of Clinical Laboratories of Tongji Hospital.

### Statistical analysis

Experimental results were expressed as mean ± standard deviation. Differences were analysed by one-way ANOVA using GraphPad Prism 7.0 (GraphPad Software Inc., CA) was used to generate graphs. *P* < 0.05 was considered statistically significant.

## Results

### Curdione attenuated bleomycin-induced pulmonary fibrosis in mice

**Fig. 2:**
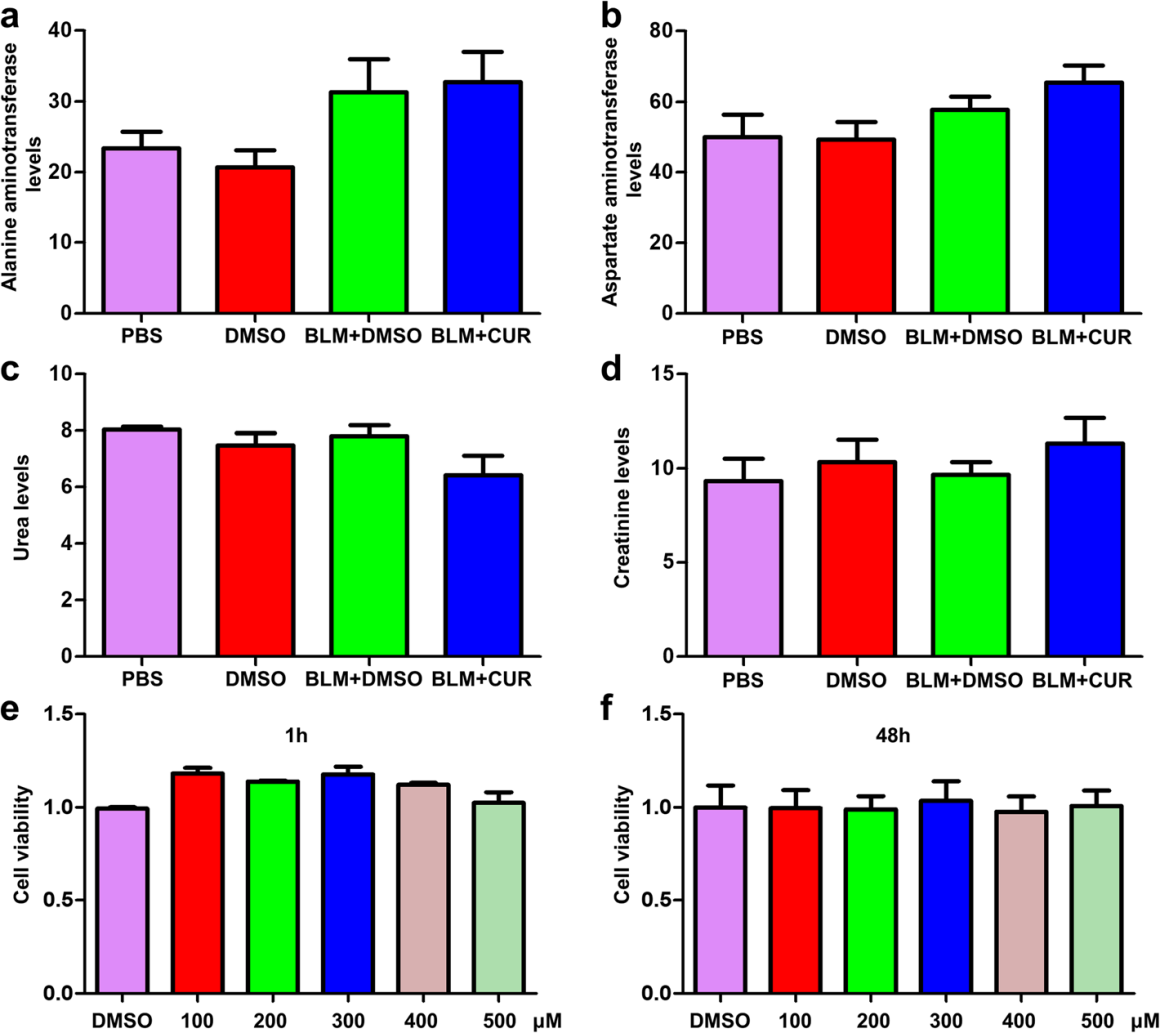
The toxic effects of curdione on the mice and cells. **a**, **b** Liver function. **c**, **d** Renal function. **e**, **f** Cells viability tested by CCK8 stimulated with different doses of curdione for 1 h and 48 h. Figures showing the data with three replications. Experimental results were expressed as mean ± standard deviation. The data were analysed by one-way ANOVA

**Fig. 3:**
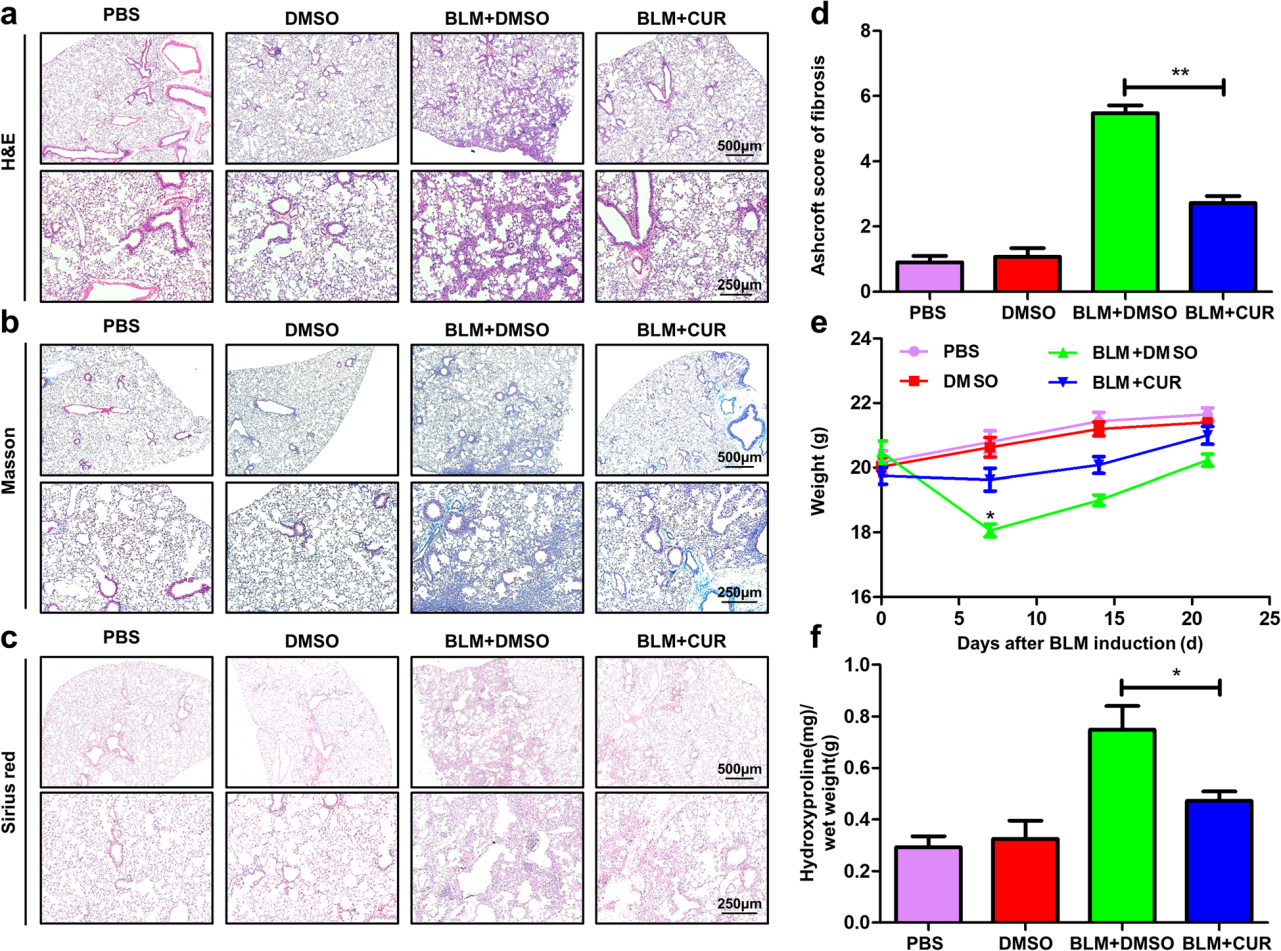
Protective effect of Curdione on pulmonary fibrosis. **a** H&E staining analysis for the severity of lung injury in mice after BLM induction. **b**, **c** Masson and Sirius red staining analysis for the severity of lung fibrosis in mice following BLM induction. Images were taken under original magnification × 40 (Up) and × 100(Down). **d** A bar graph showing the semiquantitative ashcroft scores for the severity of fibrosis. **e** Body weight change during the course of BLM-induced fibrosis. **f** The rates of hydroxyproline and lung wet weight of all mice studied. Twelve mice were included in each study group. Experimental results were expressed as mean ± standard deviation. The data were analysed by one-way ANOVA. *P < 0.05; **P < 0.01

We first sought to evaluate the effects of curdione on the development of pulmonary fibrosis. For this purpose, the mice were administered with curdione for every 2 days, followed by BLM injection as described [10]. First, the toxic effects of curdione to the mice and cells were detected. Notably, curdione did not seem to exist toxic effects on the mice, as we failed to detect perceptible differences of liver function and renal function (Fig. 2 a-d). Furthermore, curdione may not affect the cells viability, even if 500 μM of curdione lasted for 48 h, a very high dose and long enough stimulating time, was administrated to the cells (Fig. 2 e-f). Next, histopathological changes of the lungs were observed with HE, Masson and Sirius red staining to evaluate pulmonary morphology. Interestingly, compared with the BLM + DMSO groups, the BLM + CUR group showed significantly attenuated lung injury, as evidenced by H&E staining (Fig. 3 a). Significantly alleviated pulmonary fibrosis were noted in BLM + CUR group as shown by the Masson staining (Fig. 3 b) and Sirius red staining (Fig. 3 c). In particular, the Ashcroft scores of lung fibrosis was lower in BLM + CUR group compared with BLM + DMSO group (Fig. 3 d). In addition, both BLM-induced mice were undergoing significant weight loss on day 7 of BLM induction compared to mice from the control group. However, mice treated with curdione had a less weight loss than mice from the BLM + DMSO group at all examined time points (Fig. 3 e). Furthermore, from the immunohistochemical results, the infiltration level of inflammatory cells (lymphocytes and macrophages) in the curdione treatment group was also significantly lower than that in the BLM + DMSO group (Additional file 1: Figure S1).

To confirm the above data, levels of hydroxyproline, an amino acid formed upon hydrolysis of connective-tissue proteins such as collagen, were detected in all group. Indeed, compared with control group, significantly higher levels of hydroxyproline were detected in BLM-induced mice, while curdione administration attenuated BLM-induced hydroxyproline content by 1.6-fold (Fig. 3 f).

### Curdione reduced the expressions of fibrosis specific indicators induced by BLM

**Fig. 4:**
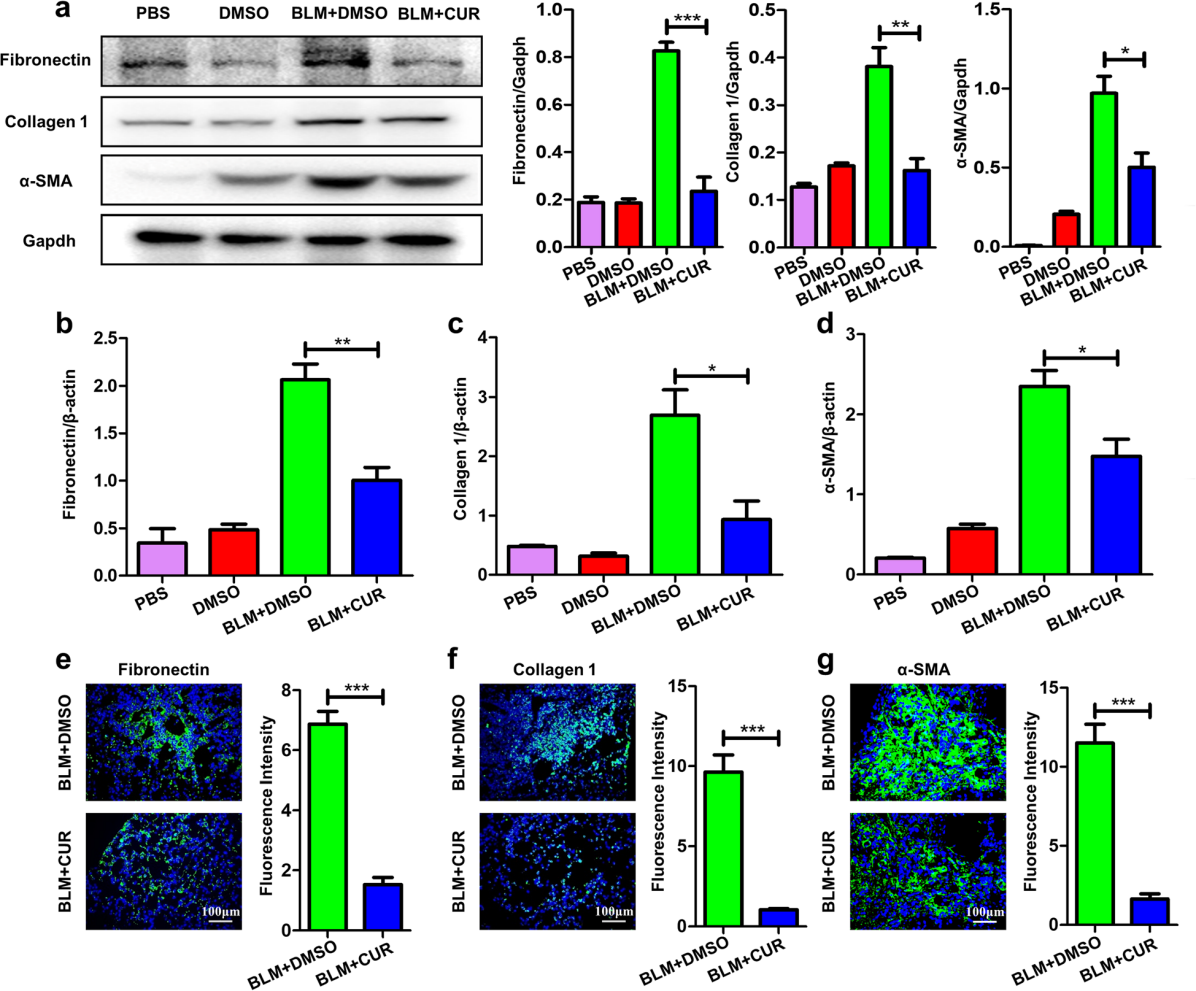
Curdione attenuated fibrotic markers expression following BLM induced. **a** Western blot analysis of fibrotic markers fibronectin, collagen 1 and α-SMA. Left panel: a representative western blot result. Right panel: a bar graph showing the mean data of all mice analyzed in each group. **b**-**d** RT-PCR analysis of fibrotic markers fibronectin, collagen 1 and α-SMA. **e** Immunostaining of fibronectin, collagen 1 and α-SMA in the lung sections. Images were taken under original magnification × 200. Twelve mice were included in each study group. Experimental results were expressed as mean ± standard deviation. The data were analysed by one-way ANOVA. *P < 0.05; **P < 0.01; ***P < 0.001

To further evaluate the effects of curdione on pulmonary fibrosis, western blot analysis of fibrotic markers was adopted in lung homogenates. Consisted with above data, the expression of fibronectin, collagen 1 and α-SMA was lower in BLM + CUR group (Fig. 4 a) than BLM + DMSO group. Similarly, RT-PCR analysis and immunofluorescence analysis of fibronectin, collagen 1 and α-SMA expression all showed consistent results (Fig. 4 b-e).

### Curdione suppressed fibroblast to myofibroblast differentiation

Given the important role that fibroblast to myofibroblast differentiation plays in the progression of pulmonary fibrosis [14], we examined differentiation of fibroblast in the lungs of the mice by western blot analysis, RT-PCR analysis and immunofluorescence. Interestingly, mice treatment with curdione displayed substantially lower α-SMA positive cells after BLM induction than the mice originating from BLM + DMSO group (Fig. 4 e), indicating that administration of curdione may reduce the differentiation of fibroblasts in vivo.

**Fig. 5:**
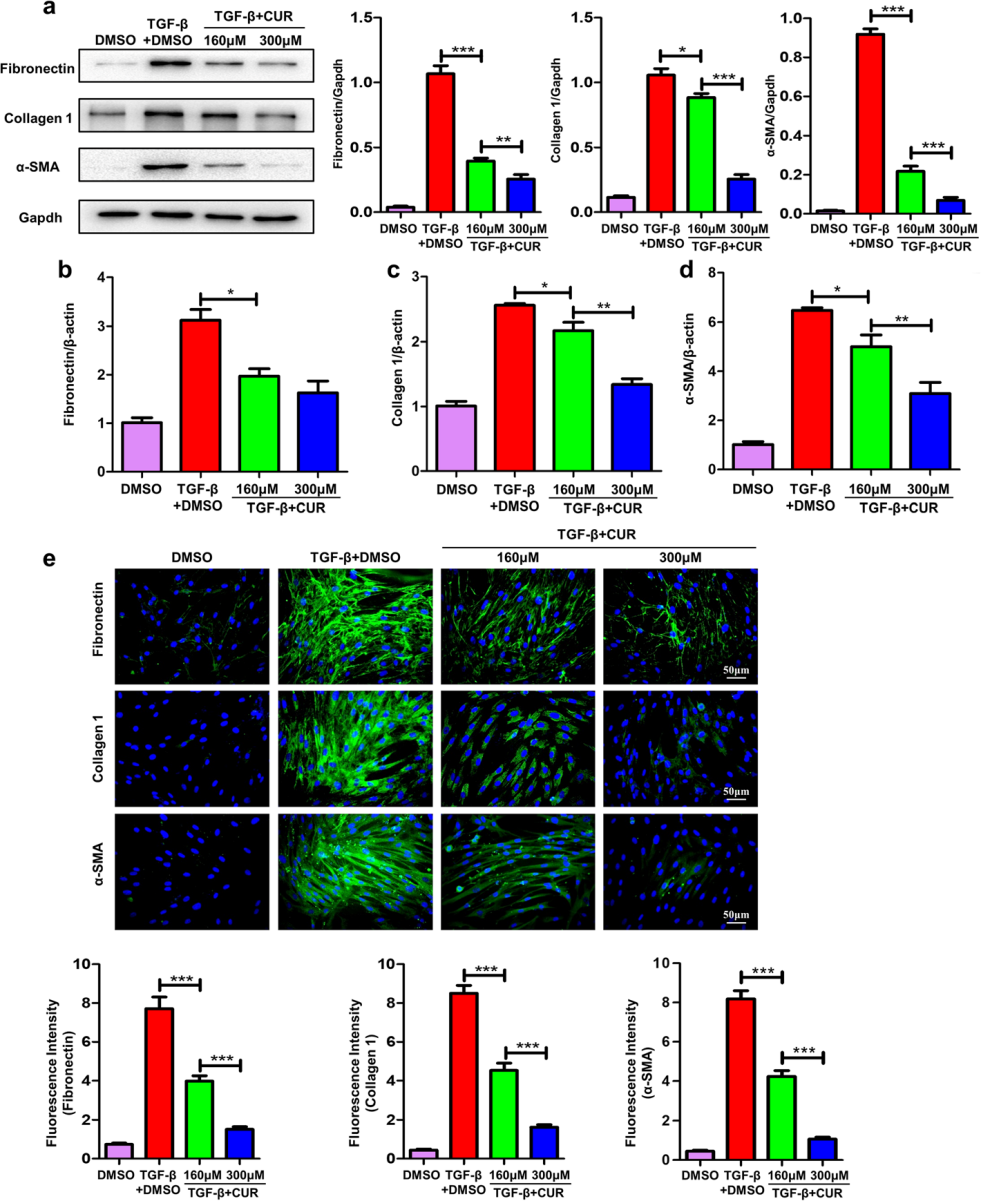
Curdione treatment inhibited fibroblast differentiation. **a** Analysis of HPFs differentiation after TGF-β stimulation. Left panel: representative western blot results for fibronectin, collagen 1 and α-SMA. Right panel: bar graphic figure for western blot. **b**-**d** RT-PCR analysis of the expression of fibronectin, collagen 1 and α-SMA. **e** Immunostaining of fibronectin, collagen 1 and α-SMA expression. Images were taken under original magnification × 400. Figures showing the data with three replications. Experimental results were expressed as mean ± standard deviation. The data were analysed by one-way ANOVA. *P < 0.05; **P < 0.01; ***P < 0.001

Based on the above observations, HPFs were used to validate the effects of curdione on fibroblast differentiation. Interestingly, administration of curdione significantly inhibited fibroblast differentiation to myofibroblast in dose dependent manner as evidenced by the significantly reduced levels of fibronectin, collagen 1 and α-SMA after TGF-β1 treatment for 48 h analyzed by western blot (Fig. 5 a), RT-PCR (Fig. 5 b - d) and immunofluorescence (Fig. 5 e).

### Curdione inhibited TGF-β/Smad3 signaling

**Fig. 6:**
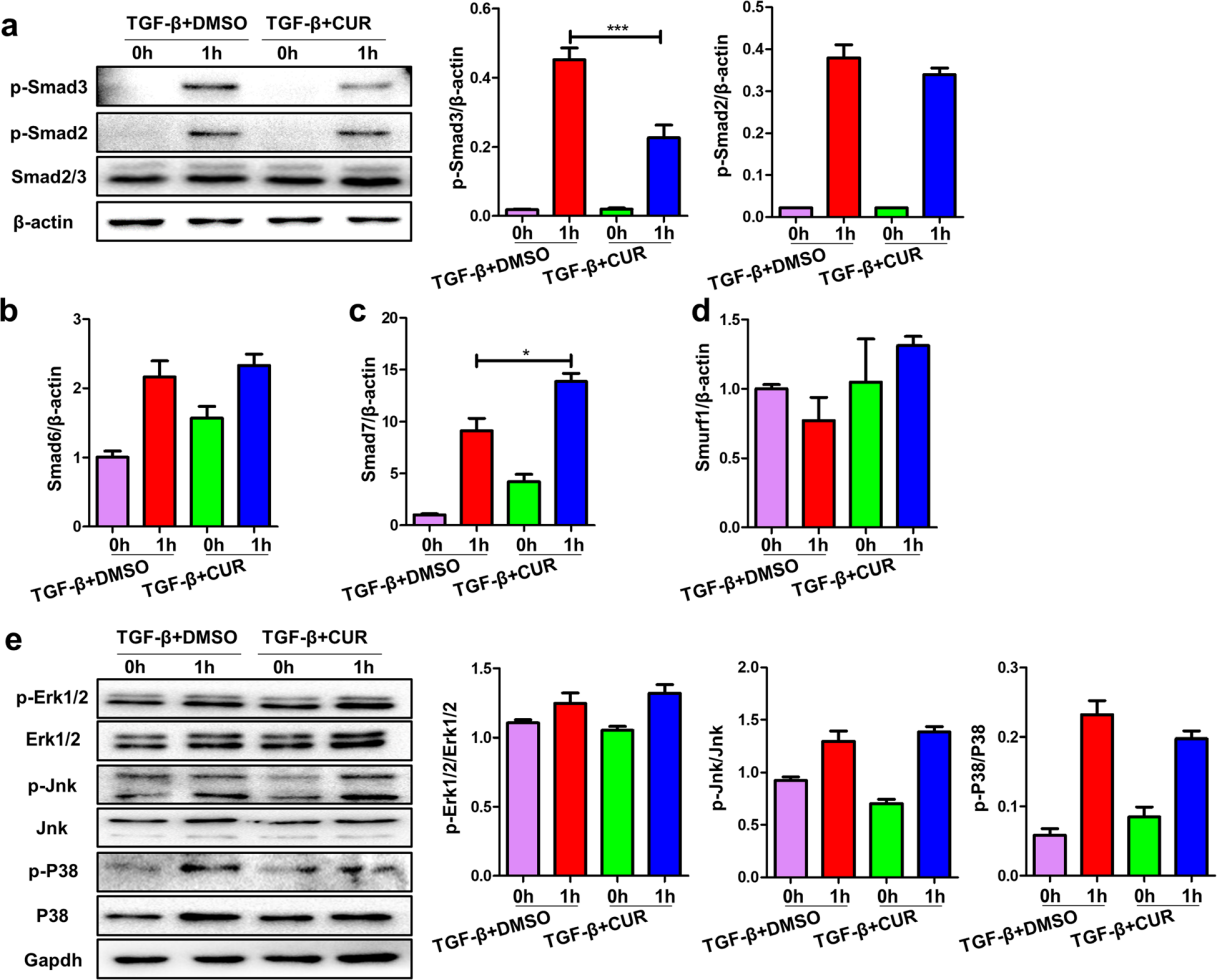
Curdione decreased the level of phosphorylated-Smad3 in TGF-β induced HPFs. **a** Left panel: a representative western blot result for p-Smad2 and p-Smad3. Right panel: bar graphic figure for western blot. **b**-**d** RT-PCR analysis of the expression of Samd6, Smad7 and Smurf1. **e** Curdione administration did not affect MAPK signaling. Figures showing the data with three replications. Experimental results were expressed as mean ± standard deviation. The data were analysed by one-way ANOVA. *P < 0.05; ***P < 0.001

TGF-β/Smad signaling has been suggested to be critical for an optimal and sustained fibroblast differentiation upon TGF-β stimulation [15]. Therefore, we examined the impact of curdione on TGF-β/Smad signaling in TGF-β1-stimulated HPFs. Interestingly, TGF-β1 treatment significantly induced Smad activation as manifested by increasing of p-Smad2 and p-Smad3 levels, while curdione administration significantly attenuated TGF-β1-induced p-Smad3 activation, but not p-Smad2 (Fig. 6 a). Furthermore, we also detected others Smad signal pathway proteins by RT-PCR (Fig. 6 b-d). Interestingly, the expression of Smad7, which could inhibit p-Smad3, was increased following curdione treatment. Therefore, we speculate that up-regulation of Smad7 is a potential intermediate mechanism for the down-phosphorylation of Smad3 by curdione. Of note, MAPK signaling are also implicated in fibroblast differentiation [16]. However, no differences were detected in terms of phosphorylated forms (p-P38, p-Jnk, p-Erk1/2) between all experimental groups after TGF-β1 induction for 1 h (Fig. 6 e).

## Discussion

In the current study, we verified the therapeutic effects of curdione on pulmonary fibrosis. Herein, our study demonstrated that curdione treatment might effectively reduce lung injury and fibrosis induced by BLM and attenuate the differentiation of fibroblast to myofibroblast. The potential mechanism of the protective effects of curdione on pulmonary fibrosis might be attributed to the inhibition of TGF-β/Smad3 signaling. Together, our study might provide a new idea for treatment of IPF.

Curdione is a kind of traditional Chinese medicine, which is extracted from the rhizome of curcuma, zedoaria. Accumulating evidence indicated that curdione has anti-inflammatory, anti-apoptotic and anti-tumor effects [4–8, 17]. However, the role of curdione on pulmonary fibrosis has been rarely reported. As the pathogenesis of pulmonary fibrosis is closely related to oxidative stress and inflammatory response, we speculate curdione may have therapeutic effects on pulmonary fibrosis. Therefore, we first investigated the effects of curdione on the development of pulmonary fibrosis by establishing a BLM-mediated mouse model.

First of all, we determined the safety of curdione in vivo by comparing the liver and renal functions of mice with or without curdione. Considering that the solvent DMSO may be toxic to some extent, we set up a pure DMSO group to eliminate interference. Interestingly, the results showed curdione had excellent safety as manifested by no obvious damages were detected on liver and renal function in mice treatment with curdione. Next, we verified the effects of curdione on pulmonary fibrosis. Curdione could significantly reduce the degree of pulmonary fibrosis in BLM-induced mice as manifested by H&E, Masson and Sirius red staining. Furthermore, after application of curdione, Ashcroft score of lung sections and hydroxyproline content in lung tissues decreased significantly. In addition, the fibrosis indicators fibronectin, collagen 1, and α-SMA all showed varying degrees of decline by western blot, RT-PCR and immunofluorescence. Administration of curdione could also weaken the effect of bleomycin on body weight loss in mice, which might reflect in part the therapeutic effects of curdione on pulmonary fibrosis.

Accumulation and persistence of fibroblast to myofibroblast differentiation executed central role in fibrotic process [1]. To further confirm the therapeutic effects of curdione on pulmonary fibrosis, HPFs were cultured, and then stimulated by TGF-β1 to promote the differentiation. Similarly, we examined the expression level of fibronectin, collagen 1 and α-SMA by western blot, RT-PCR and immunofluorescence, and the results indicated that curdione could inhibit the differentiation of fibroblast induced by TGF-β1 in a dose-dependent manner, which is consisted with previous data in vivo.

In addition to fibroblast differentiation, inflammation and oxidative stress also played a key role in IPF pathogenesis. Studies have shown that the contents of inflammatory cells (macrophages, lymphocytes, neutrophils) and fibroblasts as well as their oxidative stress levels were significantly increased in patients with pulmonary fibrosis [18]. Previous data showed curdione could attenuate the expression of LPS-activated COX-2, which might strengthen the anti-inflammatory effect of curdione [19]. Interestingly, in our study, the content of inflammatory cells in BLM + CUR group was significantly lower than that in BLM + DMSO group, which consisted with previous reports [19]. Increased production of reactive oxygen species could cause structural changes in DNA lipids and proteins, leading to structural damage to cells and tissues in the lung, thus promoting the progress of fibrosis disease [20]. In recent years, many studies have revealed the anti-oxidative stress effects of curdione. Li xing-jie et al. found that curdione could defend against oxidative damage and increase the activities of antioxidants (SO, CAT and GSH-PX) in MCAO rats [21].

Previous studies have revealed that TGF-β could induce Smad2 and Smad3 phosphorylation to promote fibroblast differentiation [22]. This discovery prompt us to focus on the impact of curdione on TGF-β/Smad signaling. Indeed, Smad signaling was intensified following TGF-β1 stimulation in HPFs. Surprisingly, curdione treatment markedly inhibited TGF-β1-induced Smad3 phosphorylation as manifested by significantly lower levels of p-Smad3 in TGF-β1 + curdione group compared with TGF-β1 group in HPFs. However, it seemed that Smad2 was not affected by curdione. Smad6, Smad7 and Smurf1 were important inhibitory molecules in the TGF-β signaling pathway, which could inhibit the phosphorylation of Smad3 [23]. Interestingly, we found curdione could increase the expression of Smad7. Therefore, we speculated that curdione mediated the inhibition of fibroblast differentiation by up-regulating Smad7 and then inhibiting the phosphorylation of Smad3. Several other signaling pathways were also found to be closely related to the differentiation of fibroblast, such as the MAPK signaling [24]. However, we failed to detect a significant difference in terms of the increase in the phosphorylated forms of P38, Jnk, and Erk1/2 with or without curdione treatment. Taken together, these results suggested that curdione might suppress fibroblast differentiation at least by inhibition of TGF-β/Smad3 signaling.

## Conclusion

In conclusion, curdione might attenuate the progress of pulmonary fibrosis and suppress the differentiation of fibroblast through TGF-β/Smad3 signaling. However, our study only demonstrated the inhibitory effect of curdione on the development of pulmonary fibrosis, further study is needed to determine whether curdione could reverse the disease in a fully established pulmonary fibrosis mouse model. Besides, more mechanisms of curdione on pulmonary fibrosis needed to be explored so that we could have fuller evidence to support the potential clinical value of the agent.

## Supplementary information

**Additional file 1: Figure S1.** Curdione attenuated the infiltration of inflammatory cells in the lung sections following BLM induced. (A) Left panel: a representative immunostaining result for F4/80 (macrophage marker). Right panel: bar graphic figure for immunostaining. (B) Left panel: a representative immunostaining result for CD3 (lymphocyte marker). Right panel: bar graphic figure for immunostaining. Images were taken under original magnification × 400. Twelve mice were included in each study group. Experimental results were expressed as mean ± standard deviation. The data were analysed by one-way ANOVA. *P < 0.05; **P < 0.01.

## Data Availability

The datasets used and/or analyzed during the current study are available from the corresponding author on reasonable request.

## Abbreviations

BLM: Bleomycin
HPFs: Human pulmonary fibroblasts
IPF: Idiopathic pulmonary fibrosis
TGF-β: Transforming growth factor-β
