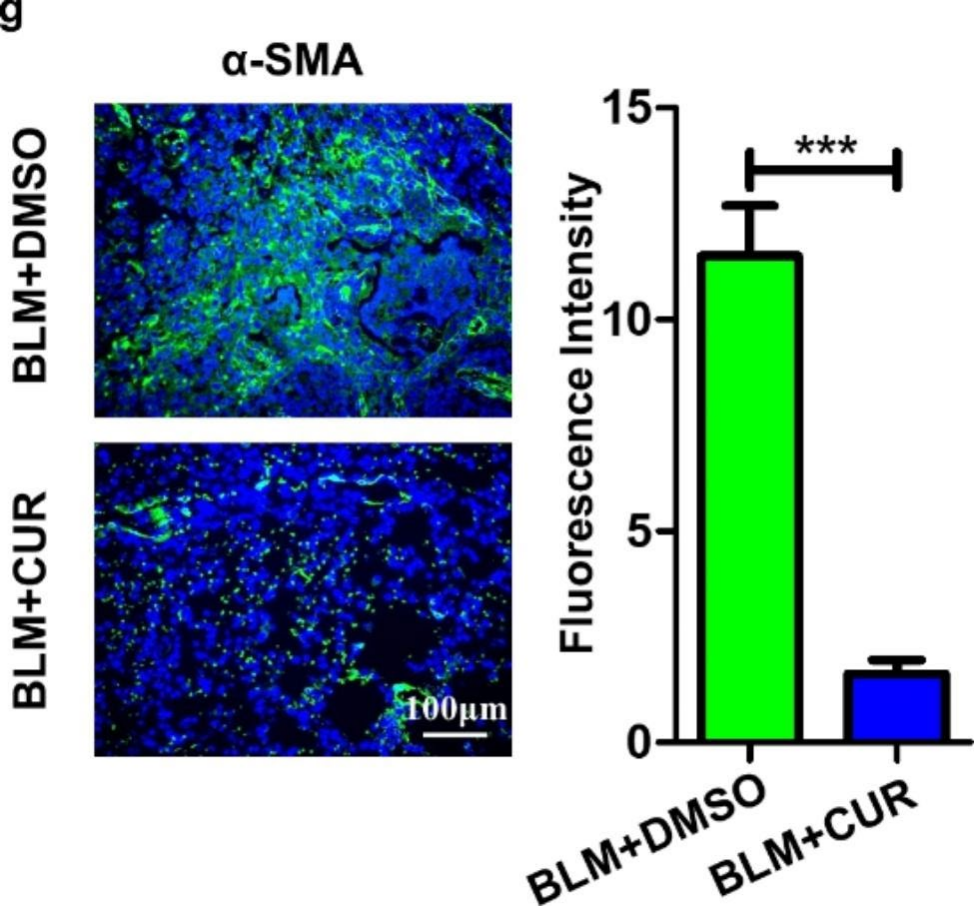# Correction to: Curdione ameliorates bleomycin-induced pulmonary fibrosis by repressing TGF-β-induced fibroblast to myofibroblast differentiation

**DOI:** 10.1186/s12931-023-02416-3

**Published:** 2023-04-17

**Authors:** Peng Liu, Kang Miao, Lei Zhang, Yong Mou, Yongjian Xu, Weining Xiong, Jun Yu, Yi Wang

**Affiliations:** 1 Department of Respiratory and Critical Care Medicine, Key Laboratory of Pulmonary Diseases of Health Ministry, Key Cite of National Clinical Research Center for Respiratory Disease, Wuhan Clinical Medical Research Center for Chronic Airway Diseases, Tongji Hospital, Tongji Medical College, Huazhong University of Sciences and Technology, 1095 Jiefang Ave, Wuhan, 430030 China; 2 Department of Respiratory Medicine, Shanghai Ninth People’s Hospital, Shanghai Jiaotong University School of Medicine, 639 Zhizaoju Lu, Shanghai, 201999 China; 3 Department of Thoracic Surgery, Tongji Hospital, Tongji Medical College, Huazhong University of Sciences and Technology, 1095 Jiefang Ave, Wuhan, 430030 China

**Correction to: Liu et al. Respiratory Research (2020) 21:58**

10.1186/s12931-020-1300-y

Following publication of the original article [1], the authors identified an error in Fig. 4g.

During the preparation of the figures in the above article, the authors regret that an error occurred during the assembly of Fig. 4g. We mistakenly put the images within the BLM + DMSO group into the BLM + CUR group. The authors confirm that these corrections do not change the result interpretation or conclusions of the article. The authors are deeply sorry and sincerely apologize for any inconvenience or misunderstanding that may have caused.

The correct version of figure is given below.