# Supplementary figures and images for: Curdione ameliorates bleomycin-induced pulmonary fibrosis by repressing TGF-β-induced fibroblast to myofibroblast differentiation
Source: Respir Res. 2020 Feb 19;21:58. doi: 10.1186/s12931-020-1300-y (PMC7031930; doi:10.1186/s12931-020-1300-y)

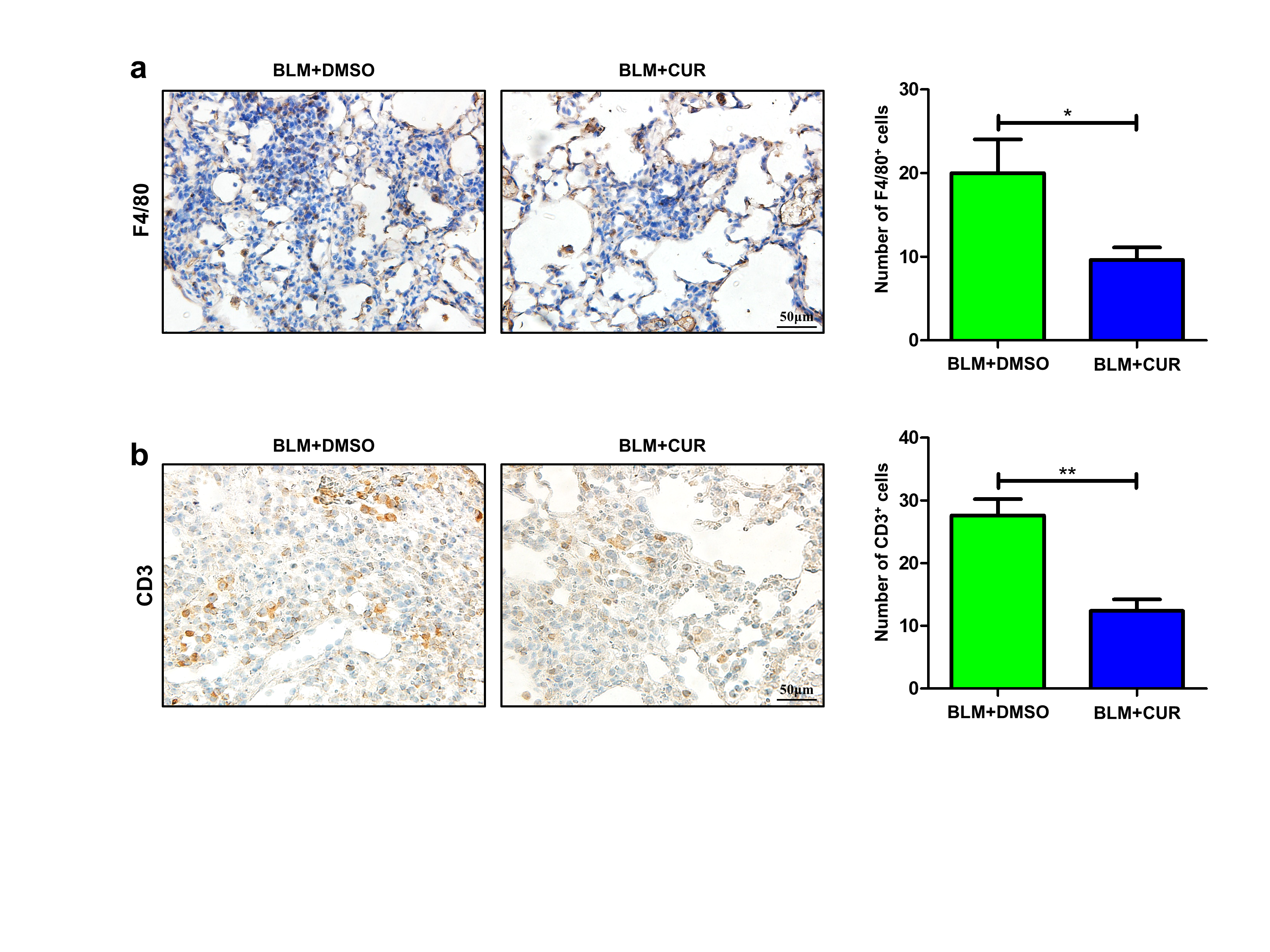

Supplement: Supplementary file 1 — Additional file 1: Figure S1. Curdione attenuated the infiltration of inflammatory cells in the lung sections following BLM induced. (A) Left panel: a representative immunostaining result for F4/80 (macrophage marker). Right panel: bar graphic figure for immunostaining. (B) Left panel: a representative immunostaining result for CD3 (lymphocyte marker). Right panel: bar graphic figure for immunostaining. Images were taken under original magnification × 400. Twelve mice were included in each study group. Experimental results were expressed as mean ± standard deviation. The data were analysed by one-way ANOVA. *P < 0.05; **P < 0.01. [file 12931_2020_1300_MOESM1_ESM.tif]
